# Associations between 24‐hour movement behavior and cognition in older adults at risk for dementia: A compositional data analysis

**DOI:** 10.1002/alz70860_100734

**Published:** 2025-12-23

**Authors:** Xiuxiu Huang, Chengfengyi Yang, Shifang Zhang, Qiaoqin Wan

**Affiliations:** ^1^ School of Nursing, Shanghai Jiao Tong University, Shanghai, Shanghai, China; ^2^ School of Nursing, Peking University, Beijing, Beijing, China

## Abstract

**Background:**

This study aims to explore the associations between the time reallocation of 24‐hour movement behaviors (24HMB) and cognition in older adults at risk for dementia.

**Method:**

This study adopted a cross‐sectional design. Community‐dwelling older adults at risk for dementia, including subjective cognitive decline and mild cognitive impairment, were recruited from community health centers and memory clinics. Global cognition was assessed by the Montreal Cognitive Assessment scale. 24HMB was measured by wrist‐worn ActiGraph for consecutive 7 days. Compositional regression model and compositional isotemporal substitution model were used to analyze the relations between 24HMB time‐use data and global cognition.

**Result:**

A total of 136 participants were included in the analysis. Compositional regression analysis suggested that the time allocation on moderate‐to‐high physical activities were positively associated with global cognition after adjusting for age, education, and gender. The compositional isotemporal substitution model revealed that replacing 10‐minute, 20‐minute, 30‐minute sedentary or sleep with moderate‐to‐high physical activities was associated with better cognition performance. No other significant results were found.

**Conclusion:**

The time‐use composition of 24HMB, particularly the time allocated on moderate‐to‐high physical activities, is associated with cognitive function in older adults at risk for dementia. Replacing sedentary, and sleep with moderate‐to‐high physical activities may induce positive effects on cognitive function.

